# Obliteration Rate of Arteriovenous Malformation by Radiosurgery

**DOI:** 10.21315/mjms-05-2025-324

**Published:** 2025-12-31

**Authors:** Sarah Atiqah Mohd Zamri, Mohd Raffiz Mohd Ali, Siti Azleen Mohamad, Abdul Rahman Izaini Ghani, Zamzuri Idris, Jafri Malin Abdullah

**Affiliations:** 1 Department of Neurosurgery, Tunku Abdul Rahman Neuroscience Institute, Hospital Kuala Lumpur, Kuala Lumpur, Malaysia; 2 Brain Behaviour Cluster, Universiti Sains Malaysia Specialist Hospital and School of Medical Sciences, Universiti Sains Malaysia, Health Campus, Kelantan, Malaysia; 3 Department of Neurosciences, School of Medical Sciences, Universiti Sains Malaysia, Health Campus, Kelantan, Malaysia

**Keywords:** radiosurgery, arteriovenous malformation, early obliteration, neurovascular disorder

## Abstract

**Background:**

The main goal of any arteriovenous malformation (AVM) intervention is to eliminate the risk of haemorrhage, which can be achieved by complete extirpation, endoluminal occlusion, or obliteration of the AVM. Radiosurgery is a minimally invasive intervention that can be performed alone or in combination with other treatments. This study aimed to determine the rate of AVM obliteration in our study population and to explore the effect of AVM characteristics, treatment mode, and treatment parameters on the obliteration rate.

**Methods:**

Three centres participating in this study obtained approval from the institutional review board and ethics committee. A total of 104 patients from among 146 were identified according to the inclusion and exclusion criteria. Data retrieved from each centre includes demographic review, AVM characteristics such as size, volume, location, eloquence, venous drainage, aneurysm association, treatment mode, and parameters. Univariate and multivariate logistic regression analyses were performed to determine the potential predictors of clinical outcome, AVM obliteration, and early obliteration.

**Results:**

Of the 104 patients who underwent radiosurgery, 45 (43.3%) were obliterated, and 88.9% were obliterated within three years. The independent predictors of AVM obliteration were low Spetzler-Martin grading (*P* = 0.005), dose > 22 Gy (*P* = 0.001), low radiosurgery-based AVM grading scale score (*P* = 0.037), low Virginia radiosurgery AVM scale score (*P* = 0.045), fraction (*P* = 0.002), and treatment mode (*P* = 0.025). Volume was the independent predictor of early obliteration (*P* = 0.013). The presence of a neurological deficit was the independent predictor of the clinical outcome (*P* = 0.018).

**Conclusion:**

Identifying predictors of good outcomes for patients who are suitable for radiosurgery is important to ensure optimal AVM treatment.

## Introduction

Arteriovenous malformation (AVM) is characterised by a complex network of abnormal vessels consisting of feeding arteries, draining veins, and a dysplastic vascular nidus that connects the arterial and venous systems without an intervening capillary bed, resulting in a low-resistance arteriovenous connection with high-flow shunting. This may lead to chronic changes in feeders, such as smooth muscle hyperplasia, thickening of the draining vein, and abnormal dilatation of vessels forming aneurysms (1). Yasargil and Houdart, in Meder et al. (2) classified AVM according to its angioarchitecture into two types: the plexiform type, with a simple network of compact or loose arteriovenous shunts, and the non-plexiform type with direct arteriovenous fistula or intranidal draining vein.

The most common age range in patients with AVM is 20 to 40 years old (1) with equal gender ratio affected (3).

Approximately 20% of the patients were asymptomatic, whereas the remaining patients presented with various symptoms. The most common symptoms in patients with AVM are intracranial haemorrhage (50%) (3, 4), seizure (27%), headache, and neurological deficit (3). Haemorrhage is the most worrying presentation as it may cause great disability to the patient.

The average risk of haemorrhage is 2% to 4% per year (1). Stapf et al. (5) divided the annual risk for haemorrhage into two groups: low risk group (0.9% per year) (superficial nidus, superficial draining vein) and high risk group (34.4% per year) (deeply located nidus, deep venous drainage). The risk of bleeding when initial bleeding occurred during pregnancy was 6% at the 1-year follow-up (6). Factors associated with risk of haemorrhage in brain AVM are large size (21% in small and 18% in large AVM), prior history of haemorrhage during pregnancy, infratentorial origin (6), deep location, deep venous drainage, and presence of berry aneurysm in feeder artery (3).

Early diagnosis of AVM is important to initiate effective treatment planning. Radiological imaging is vital for establishing diagnosis. Digital subtraction angiography (DSA) is the gold standard for diagnosing AVM. It has the highest degree of resolution in the identification of the early draining vein, AVM nidus configuration, location, and feeding arteries and provides detailed information on hemodynamics (3, 7). Although angiography is the gold standard, MRI has been shown to be an accurate substitute for evaluating AVM patency after radiosurgery (8–10). Although it has some limitations in the detection of smaller vessels (< 1 mm diameter), aneurysms, smaller AVM nidus (< 1 mm), and venous outflow anatomy, MR and MRA features have significantly improved in terms of spatial and temporal resolution and are important in treatment planning (7). Pollock et al. (10) reported a sensitivity, specificity, and negative predictive value of 80%, 100%, and 91%, respectively. O’Connor and Friedman (9) reported that MRI correctly diagnoses AVM occlusion in 82% of patients, and the obliteration agreement with angiography was 90% for AVM volumes above 2.8 cm^3^ and 70% for smaller volumes.

The management protocol that we usually refer to is based on the Spetzler–Ponce classification (SPC). Based on this protocol, radiosurgery is indicated in the following patients:

- SPC Class A, SM II, unruptured, diffuse, age > 40 years old, Pollock-flickering score ≤ 2.5;
- SP Class B, SM III, unruptured, age > 40 years old, Pollock-flickering score ≤ 2.5;
- SPC Class B, SM III, unruptured, age 20 to 40 years old, diffuse, Pollock-flickering score ≤ 2.5; and
- SPC Class B, SM III, ruptured, diffuse, age > 40 years old, Pollock-flickering score ≤ 2.5.

However, AVM management should not be restricted as per the guidelines only. It should be tailored based on the individual/patient and depends on the surgeon, lesion, and patient factors (11, 12).

In this study, we aimed to determine the clinical and radiological outcomes of patients with AVM treated with radiosurgery. We would also like to explore the effect of AVM characteristics and treatment parameters on the obliteration rate and identify the AVM obliteration predictors.

## Methods

**Figure 1:**
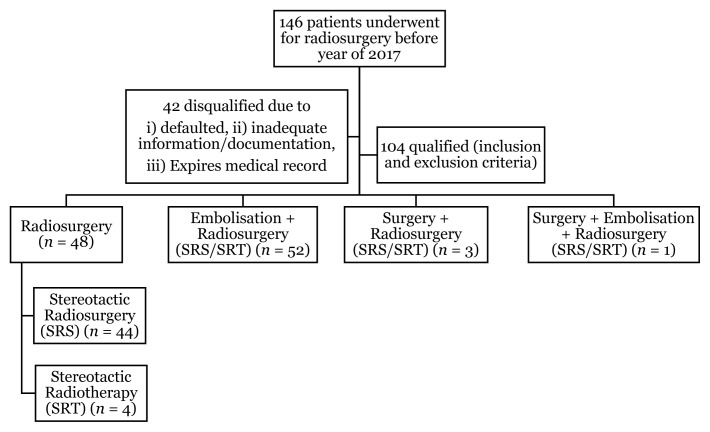
Flow diagram illustrating patient selection and treatment distribution among patients who underwent radiosurgery, including reasons for exclusion and final treatment modalities.

Of the 146 patients identified from the medical records, 42 did not have proper follow-up or adequate information for our study, and 104 were eligible to be included in this study (Figure 1). These patients were followed up from three different hospitals: Hospital Kuala Lumpur, Hospital Sungai Buloh, and Hospital Universiti Sains Malaysia. Patients or physicians at each hospital were reached out for further information or in cases where recent follow-up data were unavailable.

### Patient Populations

Three neurosurgical centres were chosen to participate in this study after obtaining approval from the institutional review board and ethics approval. Patients with AVM who underwent radiosurgery before 2017 were identified and screened for their eligibility to be included in this study according to the inclusion and exclusion criteria. Of the 146 patients identified from the medical records, 42 did not have a proper follow-up or adequate information for our study, and 104 were eligible to be included in this study. Patients or physicians at each hospital were contacted for further information in cases where recent follow-up data were unavailable.

A total of 104 patients who met this criterion were followed up at the following hospitals: Hospital Kuala Lumpur (HKL) (78 patients), Hospital Universiti Sains Malaysia (HUSM) (22 patients), and Hospital Sungai Buloh (HSB) (4 patients). A retrospective review of patients who underwent radiosurgery alone or in combination was performed. The medical records of the patients were studied at each centre, and the information was entered into the case report form before it was submitted to a computerised database. All patient identifications have been removed. The list of patients diagnosed with AVM and treated with stereotactic radiosurgery (SRS)/stereotactic radiotherapy (SRT) alone or with a combination of another modality will be identified from the clinic census. The medical records of these patients will be traced, and relevant information will be retrieved for further analysis. The collected information includes demographic, clinical, AVM characteristics, radiological, treatment mode, treatment parameters, and outcome.

In this study, AVM and its obliteration will be confirmed by MRI/MRA/MRV and/or DSA. The series of MRI/MRA/MRV and/or DSA will be reviewed, and the radiologist who reported during the event will refer to the formal report for interpretation. Patients were categorised into four groups based on their AVM status: obliterated, reduced volume, static, and increased volume. These groups will then be further divided into early and late obliteration subcategories. AVM obliteration was defined on MRI as an absence of flow voids or on angiography as an absence of abnormal arteriovenous shunting. Early obliteration occurs when the AVM is obliterated at or before 18 months after radiosurgery. Late obliteration occurs when the AVM is obliterated more than 18 months after radiosurgery.

### Radiosurgical Technique

Linear accelerator radiosurgery was performed at HKL, HUSM, and Institut Kanser Negara (IKN). However, the stereotactic frames Crown-Robert-Well were used in HUSM and a face mask (frameless-based) in HKL and IKN. Stereotactic cerebral angiography, where MR angiography (MRA) and DSA were fused and incorporated into the treatment planning. Nidus definition and contouring were performed by a neurosurgeon. Dose planning was performed by a radiation oncologist based on the AVM characteristics, the critical structure nearby, and whether there is a history of radiation therapy. The mean doses for early and late responders were 21.1 ± 2.3 Gy and 23.5 ± 8.3 Gy, respectively.

### Combination Treatment

Multidisciplinary meetings or discussions were held before the patient was offered the choice of definitive treatment. This multidisciplinary team includes distinguished neurosurgeons, oncologists, and interventional neuroradiologists. Each team member representative will give their expert opinion, and the final decision on the best treatment for the patient will be determined. The pros and cons of each choice were explained to the patient, including the risks and complications.

### Clinical and Radiological Assessment

The demographic reviews are gender and age. Clinical assessment is based on the presentation of symptoms or signs, and the outcome is graded based on the mRS score. AVM characteristics were reviewed based on size, volume, nidus location, eloquence, venous drainage, and aneurysm presence.

Volume is calculated as follows (13, 14):


Volume=(0.5)×width×length×height

or,


Volume=(3.142/6)×width×length×height

Eloquent locations include the sensorimotor, language, visual cortex, hypothalamus and thalamus, internal capsule, brainstem, cerebellar peduncles, and deep cerebellar nuclei. In terms of severity grading of AVM, it will be classified according to the Spetzler–Martin grading (15). In contrast, the additional assessment of predictors of the AVM obliteration rate includes the Pollock-flickering score (16), radiosurgery-based AVM grading scale (RBAS) (17), and Virginia radiosurgery AVM scale (VRAS) (18). The modes of treatment that we are looking at are SRS alone, SRT alone, or a combination of surgery and SRS/SRT, embolisation and SRS/SRT, surgery, embolisation, and SRS/SRT. The dose, fraction, and treatment date were identified. The date and duration of obliteration based on the date of radio-imaging shows AVM obliteration.

Radiological outcomes are based on whether the AVM nidus is obliterated, reduced in volume, static, or increased in volume. The clinical outcome is based on mRS. The patient follow-up record must be at least until > 6 months after AVM obliteration. The patients were followed up at 6 months post-radiosurgery with neuroimaging for 18 to 24 months, then neuroimaging was repeated annually. Folders will be retrieved from medical records, and the relevant information obtained will be recorded in a predesigned case report form, which will be subsequently transferred to SPSS 24 for further analysis.

### Statistical Analysis

Data are presented as mean and standard deviation for continuous variables and frequency and percentage for categorical variables. Categorical variables were statistically analysed using chi-square and Fisher’s exact tests as appropriate. The means were calculated using an independent *t*-test when the parameter fulfilled the normality criteria and the Mann–Whitney U test when the normality criteria were not fulfilled. Univariate and multivariate binary logistic regression analyses were performed to identify predictors of clinical outcome and early obliteration. Predictors of AVM obliteration were identified by univariate and multivariate multinomial logistic regression. Factors analysed in this analysis included sex, age, AVM characteristics, treatment mode with treatment parameters, duration of obliteration, and clinical outcome.

All statistical tests were two-sided and statistical significance was defined as *P* < 0.05.

## Results

### Demographic

Of the 146 patients identified from the medical records, 42 did not have a proper follow-up or adequate information for our study, and 104 were eligible to be included in this study. These patients were followed up from three different hospitals: HKL, Hospital Sungai Buloh, and HUSM. Of these 104 patients, 45 (43.3%) had their AVM totally obliterated, 50 (48.1%) had reduced in size, one (1.0%) remained the same, and eight (7.7%) had an increase in AVM size. Of the total AVMs that underwent radiosurgery, 88.9% were obliterated within three years. Table 1 summarises the patients’ demographics, AVM characteristics, and treatment parameters.

### AVM Characteristic and Grading

The mean size diameter for early and late responders was 1.463 and 1.982 cm, respectively (*P* = 0.008). The majority of patients with total AVM obliteration had AVM sizes of less than 3 cm (*P* = 0.046). The mean volume for the early responder was 1.146 cm^3^ and that for the late responder was 3.562 cm^3^ (*P* = 0.005). Volume gave a significant result with a *P*-value of 0.033, and most obliterated AVM had a volume of less than 4.00 cc (*P* = 0.014).

No significant differences were observed in the AVM nidus location (*P* = 0.490). However, in our study, only AVMs in the frontal, temporal, parietal, occipital, thalamus, and cerebellum were obliterated. AVM nidus located in the basal ganglia, brainstem, ventricle, pineal region, and corpus callosum were not completely obliterated. No significant difference was found in eloquence (*P* = 0.178), venous drainage (*P* = 0.188), grading based on the Spetzler–Martin classification (*P* =0.773), VRAS score (*P* = 0.434), RBAS score (*P* = 0.235), and Pollock-flickering score (*P* = 0.098). Only two of nine AVMs associated with aneurysms were obliterated (*P* = 1.000).

### Treatment Parameters and Outcomes

The majority of early responders had no history of prior embolisation (radiosurgery alone, 70.6%) or a history of prior embolisation (29.4%). Compared with late responders who had a history of prior embolisation (50%) and radiosurgery alone (46.4%), both results are with insignificant *P*-values. The majority of early responders received radiosurgery with doses of 22 to 24 Gy (70.6%), and others, < 18 Gy (23.5%) and 18 to 20 Gy (5.9%). Of the early responders, 94.1% had a favourable outcome (mRS 0 to 3), and of the late responders, only 71.4% had a favourable outcome. Unfavourable outcomes (mRS 4 to 6) in early and late responders were 5.9% and 28.6%, respectively.

Independent predictors of the clinical outcome in the univariate and multivariate logistic regression analyses are summarised in Table 2.

On univariate analysis, the predictors of the clinical outcome were age (odds ratio [OR] = 1.041; *P* = 0.009; 95% CI: 1.010, 1.074), presence of symptoms (*P* = 0.032), presence of neurological deficit (OR = 6.125; *P* = 0.033; 95% CI: 1.159, 32.366), VRAS score (OR = 1.529; *P* = 0.020; 95% CI: 1.069, 2.187), and short duration obliteration (OR = 1.120; *P* = 0.037; 95% CI: 1.007, 1.245). Eloquence was almost significant in univariate analysis with OR = 2.4; *P* = 0.0050, and 95% CI: 1.002, 5.751. On multivariate analysis, the presence of neurological deficit was the predictor of clinical outcome (OR = 122.353; *P* = 0.018; 95% CI: 2.313, 6473.549).

Independent predictors of the radiological outcome (AVM obliteration) in the univariate and multivariate logistic regression analyses are summarised in Table 3.

On univariate analysis, the predictors for AVM obliteration were the presence of symptoms (*P* = 0.001), size (OR = 0.542; *P* = 0.021; 95% CI: 0.323, 0.912), volume (OR = 0.928, *P* = 0.021; 95% CI: 0.864, 0.996), SM grading (OR = 0.209, *P* = 0.002; 95% CI: 0.076, 0.571), VRAS score (OR = 0.316; *P* = 0.001; 95% CI: 0.156, 0.641), RBAS score (OR = 0.457; *P* = 0.020; 95% CI: 0.236, 0.885), Pollock-flickering score (OR = 0.494; *P* = 0.025; 95% CI: 0.266, 0.916), and fractions (OR = 43.344; *P* = 0.000; 95% CI: 36.110, 52.028). On multivariate analysis, the predictors for AVM obliteration were SM grading (OR = 0.126; *P* = 0.005; 95% CI: 0.030, 0.528), dose (OR = 1.477; *P* = 0.001; 95% CI: 1.174, 1.783), RBAS score (OR = 368.64; *P* = 0.037; 95% CI: 1.434, 94849.076), VRAS score (OR = 0.328; *P* = 0.045; 95% CI: 0.110, 0.978), fraction (OR = 1600.615; *P* = 0.002; 95% CI: 14.266, 179580.124), and treatment mode (OR = 0.246; *P* = 0.025; 95% CI: 0.073, 0.835).

Independent predictors of early AVM obliteration in the univariate and multivariate logistic regression analyses are summarised in Table 4.

On univariate analysis, predictors for early AVM obliteration are volume < 2 cc (OR = 9.643; *P* = 0.043; 95% CI: 1.079, 86.214), treatment mode (*P* = 0.000) with radiosurgery only (OR = 2.978E7; *P* = 0.000), and no history of prior embolisation (OR = 1.152E7; *P* = 0.000). On multivariate analysis, the predictor of early obliteration was volume (OR = 1.989; *P* = 0.013; 95% CI: 1.153, 3.430).

## Discussion

The goals of any intervention or treatment for AVM are to reduce the frequency of seizures and improve symptomatic “vascular steal” or neurological deficits. However, the aim or objective for AVM treatment is to abolish the risk of haemorrhage, which can be achieved by complete extirpation, endoluminal occlusion, or obliteration of the AVM nidus (19, 20). Treatment choices include medical management only and/or interventional neurosurgery by microsurgery, radiosurgery, and/or embolisation. Radiosurgery is one of the least invasive AVM treatments. The goal of radiosurgery for AVM is complete nidus obliteration, thus eliminating the risk of future haemorrhage (20).

Most studies have shown AVM obliteration in 70% to 80% of AVMs, and obliteration is typically achieved within two to three years after treatment (7). The latency period between radiosurgery and total AVM obliteration is around three to five years (21). Because AVM obliteration may take up to a few years, radiosurgery may not offer protection against haemorrhage during this latency period. In contrast, surgical excision gives immediate elimination of haemorrhage risk (3). However, SRS offers a better risk-to-benefit profile owing to location (deep or eloquent area), especially for unruptured brain AVM with a small-to-moderate volume (< 12 cm^3^ in volume or < 3 cm in maximum diameter) (6).

In 2014, the ARUBA trial revealed a more than threefold increased risk of stroke and death after the initiation of interventional therapy (neurosurgery, embolisation, or stereotactic radiotherapy) and proved that medical management alone is superior to the combination of medical management and interventional therapy for the prevention of death or stroke in patients with unruptured brain AVM (22). However, in 2019, Karlsson et al. (23) re-evaluated the incidence of stroke between medical treatment and radiosurgery in 1351 ARUBA-eligible patients and showed a similar stroke incidence for the first five years, but increasing in the medical treatment group after five years. Therefore, interventional therapy has become more popular, well-developed, and advanced in the last decade.

Our results of radiosurgery treatment involving 104 patients with AVMs from three different centres indicated that 43.3% achieved complete obliteration of the AVM, while 48.1% experienced a reduction in size. Additionally, 1.0% of the cases remained static, and 7.7% showed an increase in size. The literature reported that the average duration of AVM obliteration by SRS is within one to three years (24). Our record on the obliteration rate in three years is 88.9%, which is comparable with the NASSAU study, which showed 76.4% obliteration in three years (23) and Yahya et al. (25), 74.5%. However, these data are not sub-analysed based on grading. Our sample is more generalised; however, most of them are from Spetzler–Martin grades I, II, and III. Ding et al. conducted a series of studies on radiosurgery treatment in patients with AVM according to the Spetzler–Martin grading. Based on the Ding et al. (19) series, the favourable outcome in grades I and II is 46%, and that in grades III and IV is 54%. On subanalyses of the cumulative obliteration rate in unruptured low-grade AVM (SMI and II), the favourable outcome is 76%. Actual obliteration rates were 66% and 80% at five and 10 years, respectively (26).

For SMIII, the obliteration rate was 62%, with the actuarial obliteration rate after radiosurgery being 37.3% at three years, 62.7% at five years, 71.6% at seven years, and 78.3% at 10 years (27). SRS in SM IV and V (high grade) gave a less favourable outcome with AVM obliteration of 26.2%. The actuarial obliteration rates at 3, 7, 10, and 12 years were 15%, 34%, 37%, and 42%, respectively (28). Meder et al. (2) reported that obliteration of AVM by SRS can be as early as within 12 months in 29% to 52% of cases. Therefore, we performed analyses on early vs. late responders on obliteration of AVM, which showed that in our study, there were 37.8% early responders and 62.2% late responders (104 patients). Cohen-Inbar et al. (20) reported in their study on 1398 patients that 14.2% had early obliteration and 85.8% had late obliteration. Table 5 shows a summary of predictors of clinical outcome and AVM obliteration after radiosurgery (univariate and multivariate analyses).

### Volume

Our independent predictor of AVM obliteration and early AVM obliteration is the volume. Univariate analysis showed that a volume less than 2 cm^3^ is a significant predictor of early obliteration. Most previous studies have reported that low-volume is the most significant predictor of obliteration. However, our significant volume as a predictor is very low compared with other studies. Vlaskou et al. (3) reported an obliteration rate of 77% in AVM volumes of 10 to 15 cm^3^ and 25% in volumes> 15 cm^3^. Friedman et al. (29) reported complete obliteration in 81% of AVM with volume 1 to 4 cc, 89% in 4 to 10 cc and 69% with volume > 10 cc. Meder et al. (2) reported that low-volume AVM is a factor that contributes to good response.

### Size

Smaller AVM seems to be a known predictor for favourable outcome with an early obliteration rate (6, 30). Meder et al. (2) reported a good radiosurgery response in the AVM nidus with maximal length of 2.5 cm. Our univariate analysis showed that a nidus size of less than 2 cm is an independent predictor for AVM obliteration.

### Doses

In our study, the dose was not statistically significant in early obliteration. However, a dose of > 22 Gy was a significant predictor in the statistical analysis of AVM obliteration. Cohen-Inbar et al. (20) reported that a higher dose of > 24 Gy is a predictor for early obliteration of AVM, and Kano et al. (30) reported that a lower marginal dose is a factor for a lower obliteration rate.

### Previous Embolisations (SRS and Radiosurgery)

Many studies have reported that embolisation has the least success for total obliteration. It must be combined with another modality, either surgery or radiosurgery. Embolisation was performed before radiosurgery to reduce the size of the AVM or obliterate the intranidal aneurysm and reduce the risk of haemorrhage during the latency period after radiosurgery (3). However, studies have shown that radiosurgery alone shows better obliteration rates than when combined with embolisation (63% vs. 48%).

This is probably due to differences in the pre-embolisation of AVM angioarchitecture, and due to recanalisation of previous occluded vessels leading to enlargement of nidus (3), embolisation-induced angiogenesis, desensitisation to radiation, and increased difficulty of radiosurgical targeting due to artefacts from embolic material agent artefact, thereby reducing the efficacy of radiosurgery (4).

Our study shows that a history of embolisation before radiotherapy is a predictor of a poor response to total obliteration, and treatment with radiosurgery alone has been shown to have a better response to obliteration than a history of embolisation. Ding et al. (4) reported that the actuarial obliteration rates of low-grade AVMs treated with embolisation before radiosurgery were 24%, 34%, 49%, and 55% at 3, 5, 7, and 10 years, respectively. For low-grade AVMs without prior embolisation, the actuarial obliteration rates were 45%, 74%, 81%, and 87%, respectively (4). Kato et al. (6) reported that the obliteration rate of endovascular embolisation alone does not exceed 60%. In a study by Singfer et al. (31), the standard occlusion rate was only 29.8% with endovascular embolisation alone, but a high overall occlusion rate of 73.7% in combination with SRS. Inaccurate definition of nidus and recanalisation due to a history of previous embolisation prior to radiosurgery are also factors of lower obliteration rate (30).

### AVM Associated with Aneurysm

The absence of association with aneurysm seems to be one of the factors for a favourable outcome in the Kato et al. (6) study. A total of nine out of 104 patients with AVM in our study were associated with an aneurysm. Only two out of nine patients with AVM were obliterated (one early and one late). However, this result was statistically insignificant, most likely due to the small sample size. Kato et al. (6) also recommended that in the presence of feeder artery or intranidal aneurysm associated with AVM, the aneurysm should be treated first in view of the higher risk of haemorrhage (32).

Other factors that were excluded in our study but reported in the literature are plexiform AVM showing better response with radiosurgery (2), supratentorial location (6), and younger age. Kemeny et al. (33) reported that younger age seems to give better response—75% favourable in those under 20 years old, 45% favourable between 20 and 40 years old, and 25% favourable in those aged above 40 years old.

### Study Limitations

The most significant limitation of this study was the sample size. Because of the small sample size, certain results are invalid, and it is difficult to obtain statistically significant results despite being descriptively significant.

Many patients were disqualified from the study due to multifactorial reasons. Some patients defaulted on follow-up because of difficulty in mobilising or attending clinic follow-ups due to neurological deficits, poor social support, and inadequate health education. Some patients could not abide by a proper follow-up routine because of transportation problems.

Poor documentation, especially in very old medical reports, leads to improper assessment judgements, thereby resulting in inadequate information and disqualification from participation in this study.

Subgroup analyses could not be performed due to the need for larger samples.

In addition, in certain cases, an earlier or more consistent interval of repeat imaging could not be performed due to multiple reasons. This may lead to late documentation of obliteration when this patient has a high possibility of having their AVM obliterated earlier. Therefore, this could lead to the underestimation of the real-time probability of early obliteration.

## Conclusion

Most patients diagnosed with AVM are young and have a long life expectancy. Therefore, a longer follow-up study is needed to look for outcomes and late complications, such as cyst formation, radiation-induced changes, or neoplasms. In the future, further studies should be conducted regarding adverse events or side effects of radiosurgery treatments, such as symptomatic radiation changes, post-radiosurgery haemorrhage, or permanent neurological morbidity. The formation of an AVM board with a team of neurosurgeons who specialise in AVM surgery, embolisation, and radiation therapy using multimodal assessment is needed. Careful selection of patients for radiosurgery may benefit from early obliteration and avoid complications.

## Figures and Tables

**Table 1:** Patient’s demographic characteristics, AVM characteristics, and treatment parameters

Parameters	Early obliteration	Late responders	*P*-value
Total number of patients treated with radiosurgery = 104	45 (43.3%)		

Total number of patients with AVM obliteration	17 (37.8%)	28 (62.2%)	

Mean obliteration duration	12.65 months	35.75 months	0.000

Male : Female	9 : 8 (52.9% : 47.1%)	15 : 13 (53.6% : 46.4%)	1.000

Mean age (years)	29.53	27.2	0.531

Age			
< 18	5 (29.4%)	8 (28.6%)	0.376
19 to 65	12 (70.6%)	20 (71.4%)	0.483
> 65	0	0	

Treatment mode			
Radiosurgery (SRS or SRT)	12 (70.6%)	13 (46.4%)	0.050
Prior embolisation	5 (29.4%)	14 (50%)	0.165
Prior surgery	0	1 (3.6%)	

Symptoms/Presentations			
Headache	0	3 (6.7%)	
Seizure	2 (11.8%)	7 (20.0%)	
Haemorrhage	1 (5.9%)	2 (6.7%)	0.231
Neurological deficit	1 (5.9%)	4 (11.1)	
More than one symptom as above	13 (76.5%)	12 (55.6%)	

Radiological conformation			
MRI/MRA/MRV	2 (11.8%)	7 (25.0%)	
DSA	6 (35.3%)	10 (35.7%)	0.504
Both	9 (52.9%)	11 (39.3%)	

Associated with aneurysm	1 (5.9%)	1 (3.6%)	1.000
* 9/104 AVM cases associated with aneurysm; in only 2 cases AVM was obliterated			

Mean size/diameter (cm)	1.463	1.982	**0.008**

Size			
< 3 cm	16 (94.1%)	23 (82.1%)	**0.010**
3 to 6 cm	1 (5.9%)	4(14.3%)	0.287
> 6	0	1 (3.6%)	

Mean volume (cm^3^)	1.146	3.562	**0.005**

Volume			**0.033**
< 2.00 cc	15 (88.2%)	14 (50%)	**0.024**
2.01 to 4.00 cc	1 (5.9%)	5 (17.9%)	0.306
> 4.00 cc	1 (5.9%)	9 (32.1%)	0.265

Location			
Supratentorial	16 (94.1%)	26 (92.9%)	1.000
Infratentorial	1 (5.9%)	2 (7.1%)	

AVM nidus location			
Frontal	5 (29.4%)	4 (14.3%)	0.490
Temporal	2 (11.8%)	9 (32.1%)	
Parietal	5 (29.4%)	7 (25%)	
Occipital	2 (11.8%)	1 (3.6%)	
Thalamic	2 (11.8)	5 (17.9)	
Cerebellum	1 (5.9%)	2 (7.1%)	
Basal ganglia	0	0	No cases with total AVM obliteration (only reduction in size)
Brainstem	0	0	
Ventricle	0	0	
Pineal region	0	0	
Corpus callosum	0	0	

Eloquence			
Yes	6 (35.3%)	17 (60.7%)	0.178
No	11 (64.7%)	11 (39.3%)	

Venous drainage			
Superficial	8 (47.1%)	20 (71.4%)	0.188
Deep	9 (52.9%)	8 (28.6%)	

SM classification			
I	6 (35.3%)	8 (28.6%)	0.773
II	7 (41.2%)	10 (35.7%)	
III	4 (23.5%)	9 (32.1%)	
IV	0	1 (3.6%)	
V	0	0	

VRAS score			0.680
0	2 (11.8%)	7 (25%)	
1	9 (52.9%)	10 (35.7%)	
2	5 (29.4%)	8 (28.6%)	
3	1 (5.9%)	2 (7.1%)	
4	0	1 (3.6%)	

RBAS			
< 1	11 (64.7%)	15 (53.6%)	0.235
1 to 1.8	6 (35.3%)	7 (25.0%)	
1.8 to 2.5	0	4 (14.3%)	
≥ 2.5	0	2 (7.1%)	

Pollock-flickering score			
< 1.0	12 (70.6%)	20 (71.4%)	0.098
1.01 to 1.5	4 (23.5%)	1 (3.6%)	
1.51 to 2.00	1 (5.9%)	4 (14.3%)	
> 2.01	0	3 (10.7%)	

Associated with aneurysm *	1 (5.9%)	1 (3.6%)	1.000

Radiosurgery			0.281
SRS	17 (100%)	24 (85.7%)	
SRT	0	4 (14.3 %)	

Dose (all patients who underwent radiosurgery = SRS and SRT)			
< 18 Gy	4 (23.5%)	5 (17.9%)	0.112
18 to 20 Gy	1 (5.9%)	6 (21.4%)	
20 to 22 Gy	0	2 (7.1%)	
22 to 24 Gy	12 (70.6%)	11 (39.3%)	
> 24 Gy	0	4 (14.3%)	

All patients who underwent SRS (*n* = 41)			
< 18 Gy	4 (23.5%)	5 (20%)	0.198
18 to 20 Gy	1 (5.9%)	6 (25.0%)	
20 to 22 Gy	0	2 (8.3%)	
22 to 24 Gy	12 (70.6%)	11 (45.8%)	

Fraction			0.264
1	17 (100%)	24 (85.7%)	
5	0	3 (10.7%)	
11	0	1 (3.6%)	

mRS			
0 to 3	16 (94.1%)	20 (71.4%)	0.144
4 to 6	1 (5.9%)	8 (28.6%)	

The mean duration for early and late responders was 12.65 and 35.75 months, respectively. No significant differences were found between genders for early and late responders (*P* = 1.000), mean age (*P* = 0.531), and age in groups (< 18 years old, *P* = 0.376 and 19 to 65 years old, *P* = 0.483).

**Table 2:** Independent predictors of the clinical outcome

Potential factors	Univariate predictors			Multivariate predictors		

	Odds ratio – exp (B)	*P*-value	95% CI	Odds ratio – exp (B)	*P*-value	95% CI
Gender				Insignificant factor		
Male vs. female	1.003	0.994	0.442, 2.280			

Age	1.041	**0.009** *	1.010, 1.074	Insignificant factor		

Symptoms/presentations		0.032*			0.207	
Headache	0.583	0.532	0.107, 3.168	8.208	0.196	0.339, 198.956
Seizure	0.368	0.106	0.110, 1.236	0.032	0.575	0.000, 5413.535
Haemorrhage	0.219	0.166	0.025, 1.878	0.000	0.999	0.000
Neurological deficit	6.125	**0.033** *	1.159, 32.366	122.353	**0.018** *	2.313, 6473.549

Size	1.324	0.055	0.994, 1.765	Insignificant factor		

Volume	1.014	0.246	0.990, 1.039	Insignificant factor		

Location				Insignificant factor		
Supratentorial vs. infratentorial	3.667	0.233	0.433, 31.077			

Nidus location		0.716		Insignificant factor		
Frontal	0.077	0.075	0.005, 1.296			
Temporal	0.179	0.196	0.013, 2.425			
Parietal	0.361	0.425	0.030, 4.418			
Occipital	0.000	0.999	0.000			
Cerebellum	0.125	0.154	0.007, 2.176			
Corpus callosum	0.000	0.999	0.000			
Thalamus	0.143	0.184	0.008, 2.517			
Basal ganglia	8.077 × 10^8^	0.999	0.000			
Brainstem	8.077 × 10^8^	1.000	0.000			

Eloquence				Insignificant factor		
Yes vs. no	2.4	0.05	1.002, 5.751			

Venous drainage				Insignificant factor		
Superficial vs. deep	1.010	0.980	0.440, 2.322			

SM grading	1.477	0.107	0.920, 2.371	Insignificant factor		

VRAS	1.529	**0.020** *	1.069, 2.187	Insignificant factor		

RBAS	1.245	0.102	0.957, 1.621	Insignificant factor		

Pollock	1.294	0.072	0.977, 1.712	Insignificant factor		

Aneurysm				Insignificant factor		
Yes vs. no	1.733	0.436	0.434, 6.918			

ModeRx	0.561			Unstable factor, thus was not included in multivariate analysis		
Radiosurgery only (SRS or SRT)	0.000	1.000	0.000			
Prior embolisation	0.000	1.000	0.000			
Prior surgery	0.000	1.000	0.000			

Dose	0.969	0.324	0.911, 1.031	Insignificant factor		

Fractions	0.940	0.559	0.763, 1.158	Insignificant factor		

Obliteration duration	1.120	**0.037** *	1.007, 1.245	1.157	0.060	0.994, 1.347

* Significant predictor (*P* < 0.05)

**Table 3:** Independent predictor of the radiological outcome (AVM obliteration)

Potential factors	Univariate			Multivariate		

	Odds ratio – exp (B)	*P*-value	95% CI	Odds ratio – exp (B)	*P*-value	95% CI
Gender				Insignificant factor		
Male vs. female	0.686	0.633	0.146, 3.22			

Age	1.028	0.405	0.964, 1.096	Insignificant factor		

Symptom/presentations		**0.001** *		Insignificant factor		
Headache	0.600	0.684	0.051, 7.012			
Seizure	1.800	0.613	0.184, 17.567			
Haemorrhage	0.600	0.684	0.051, 7.012			
Neurological deficit	1 × 10^8^	**0.000** *	24017347.62, 4.168 × 10^8^			

Size	0.542	**0.021** *	0.323, 0.912	1.037	0.928	0.476, 2.258

Volume	0.928	**0.039** *	0.864, 0.996	0.692	0.055	0.475, 1.008

Location				Insignificant factor		
Supratentorial vs. infratentorial	4.607	0.128	0.642, 33.906			

Niduslocation	Insignificant factor			Insignificant factor		
Frontal						
Temporal						
Parietal						
Occipital						
Cerebellum						
Corpus callosum						
Thalamus						
Basal ganglia						
Brainstem						

Eloquence				Insignificant factor		
Yes vs. no	0.149	0.087	0.017, 1.315			

Venos drainage				Insignificant factor		
Superficial vs. deep	2.745	0.203	0.581, 12.973			

SM grading	0.209	**0.002** *	0.076, 0.571	0.126	**0.005**	0.030, 0.528

VRAS	0.316	**0.001** *	0.156, 0.641	0.328	**0.045** *	0.110, 0.978

RBAS	0.457	**0.020** *	0.236, 0.885	368.864	**0.037** *	1.434, 94849.076

Pollock	0.494	**0.025** *	0.266, 0.916	0.088	0.235	0.002, 4.855

Aneurysm						
Yes vs. no	0.140	0.071	0.016, 1.184			

ModeRx		1.000		0.246	**0.025**	0.073, 0.835
SRS or SRT	4.444	1.000	0.000			
Prior embolisation	0.563	1.000	0.000			
Prior surgery	0.178	1.000	0.000			

Dose	1.230	0.140	0.934, 1.620	1.477	**0.001**	1.174, 1.783

Fractions	43.344	**0.000** *	36.110, 52.028	1600.615	**0.002** *	14.266, 179580.124

Obliteration duration	The factor is uncertain					

* Significant predictor (*P* < 0.05)

**Table 4:** Independent predictors of early obliteration of AVM

Potential factors	Univariate			Multivariate		

	Odds ratio – exp (B)	*P*-value	95% CI	Odds ratio – exp (B)	*P*-value	95% CI
Gender						

Male vs. female	1.026	0.967	0.307, 3.342			

Age	0.987	0.521	0.947, 1.028			

Symptom/presentation		0.500				
Headache	1750097746	0.999	0.000			
Seizure	3.792	0.137	0.655, 21.961			
Haemorrhage	2.167	0.548	0.173, 27.075			
Neurodeficit	4.333	0.217	0.423, 44.428			

Size	2.056	0.074	0.933, 4.533			

Volume		**0.059**		1.989	0.013^*^	1.153, 3.430
< 2.00 cc vs. > 4 cc	0.104	**0.043**	0.012, 0.927			
2 to 4 cc vs. > 4 cc	0.556	0.699	0.028, 10.933			

Location						
Supratentorial vs. infratentorial	1.213	0.870	0.103, 14.696			

Niduslocation		0.537				
Frontal vs. thalamus	0.320	0.288	0.039, 2.618			
Temporal vs. thalamus	1.800	0.608	0.191, 16.980			
Parietal vs. thalamus	0.560	0.570	0.076, 4.144			
Occipital vs. thalamus	0.200	0.278	0.011, 3.661			
Cerebellum vs. thalamus	0.800	0.880	0.044, 14.643			

Eloquence						
Yes vs. no	2.833	0.103	0.811, 9.898			

Venos drainage						
Superficial vs. deep	2.813	0.107	0.800, 9.882			

SM grading	1.395	0.381	0.662, 2.936			

VRAS	0.990	0.977	0.520, 1.888			

RBAS	1.867	0.287	0.591, 5.896	0.066	0.057	0.004, 1.082

Pollock	1.716	0.337	0.570, 5.166			

Aneurysm						
Yes vs. no	0.593	0.718	0.035, 10.142			

ModeRx		**0.000**				
SRS or SRT vs. prior surgery	2.978E7	0.000	8.217E6, 1.079E8			
Prior embolisation vs. prior surgery	1.152E7	0.000	1.152E7, 1.152E8			

Dose	1.081	0.274	0.941, 1.242			

Fractions	147.084	0.999	0.000			

**Table 5:** Predictors of clinical outcome and AVM obliteration after radiosurgery

Predictors	Clinical outcome		AVM obliteration		Early obliteration	

	Univariate	Multivariate	Univariate	Multivariate	Univariate	Multivariate
Age	√					

Symptoms/presentation	√		√			

Neurological deficit	√	√	√			

Size < 2 cm			√			

Volume			√		√	√

SM grading			√			

VRAS score	√		√	√		

RBAS score			√	√		

Pollock-flickering score			√			

Association with aneurysm						

Treatment mode				√	√	
Radiosurgery alone				√	√	
Prior embolisation				√	√	

Dose (> 22 Gy)				√	√	

Fraction			√	√		

Obliteration duration	√					

## References

[1] Gavin CG, Kitchen ND, Winn HR (2017). Chapter 400: pathobiology of true AVM. Youmans Neurological Surgery.

[2] Meder JF, Oppenheim C, Blustajn J, Nataf F, Merienne L, Lefkoupolos D (1997). Cerebral arteriovenous malformations: the value of radiologic parameters in predicting response to radiosurgery. AJNR Am J Neuroradiol.

[3] Vlaskou Badra E, Ermiş E, Mordasini P, Herrmann E (2018). Radiosurgery and radiotherapy for arteriovenous malformations: outcome predictors and review of the literature. J Neurosurg Sci.

[4] Ding D, Yen CP, Xu Z, Starke RM, Sheehan JP (2014). Radiosurgery for low-grade intracranial arteriovenous malformations. J Neurosurg.

[5] Stapf C, Mast H, Sciacca RR, Choi JH, Khaw AV, Connolly ES (2006). Predictors of hemorrhage in patients with untreated brain arteriovenous malformation. Neurology.

[6] Kato Y, Dong V, Chaddad F, Takizawa K, Izumo T, Fukuda H (2019). Expert consensus on the management of brain arteriovenous malformations. Asian J Neurosurg.

[7] Derdeyn CP, Zipfel GJ, Albuquerque FC, Cooke DL, Feldmann E, Sheehan JP (2017). Management of brain arteriovenous malformations: a scientific statement for healthcare professionals from the American Heart Association/American Stroke Association. Stroke.

[8] Lee CC, Reardon MA, Ball BZ, Chen CJ, Yen CP, Xu Z (2015). The predictive value of magnetic resonance imaging in evaluating intracranial arteriovenous malformation obliteration after stereotactic radiosurgery. J Neurosurg.

[9] O’Connor TE, Friedman WA (2013). Magnetic resonance imaging assessment of cerebral arteriovenous malformation obliteration after stereotactic radiosurgery. Neurosurgery.

[10] Pollock BE, Kondziolka D, Flickinger JC, Patel AK, Bissonette DJ, Lunsford LD (1996). Magnetic resonance imaging: an accurate method to evaluate arteriovenous malformations after stereotactic radiosurgery. J Neurosurg.

[11] MKaMK M, Winn HR (2017). Therapeutic decision making in the management of arteriovenous malformations of the brain. Youmans Neurological Surgery.

[12] Spetzler RF, Ponce FA (2011). A 3-tier classification of cerebral arteriovenous malformations. J Neurosurg.

[13] Söderman M, Karlsson B, Launnay L, Thuresson B, Ericson K (2000). Volume measurement of cerebral arteriovenous malformations from angiography. Neuroradiology.

[14] Conger A, Kulwin C, Lawton MT, Cohen-Gadol AA (2015). Diagnosis and evaluation of intracranial arteriovenous malformations. Surg Neurol Int.

[15] Spetzler RF, Martin NA (1986). A proposed grading system for arteriovenous malformations. J Neurosurg.

[16] Pollock BE, Flickinger JC (2008). Modification of the radiosurgery-based arteriovenous malformation grading system. Neurosurgery.

[17] Pollock BE, Storlie CB, Link MJ, Stafford SL, Garces YI, Foote RL (2017). Comparative analysis of arteriovenous malformation grading scales in predicting outcomes after stereotactic radiosurgery. J Neurosurg.

[18] Starke RM, Yen CP, Ding D, Sheehan JP (2013). A practical grading scale for predicting outcome after radiosurgery for arteriovenous malformations: analysis of 1012 treated patients. J Neurosurg.

[19] Ding D, Starke RM, Kano H, Mathieu D, Huang PP, Kondziolka D (2017). Stereotactic radiosurgery for ARUBA (A Randomized Trial of Unruptured Brain Arteriovenous Malformations)-eligible Spetzler-Martin Grade I and II arteriovenous malformations: a multicenter study. World Neurosurg.

[20] Cohen-Inbar O, Starke RM, Paisan G, Kano H, Huang PP, Rodriguez-Mercado R (2017). Early versus late arteriovenous malformation responders after stereotactic radiosurgery: an international multicentre study. J Neurosurg.

[21] Feghali J, Huang J (2020). Updates in arteriovenous malformation management: the post-ARUBA era. Stroke Vasc Neurol.

[22] Mohr JA, Parides MK, Stapf C, Moquete E, Moy CS, Overbey JR (2014). Medical management with or without interventional therapy for unruptured brain arteriovenous malformations (ARUBA): a multicentre, non-blinded, randomised trial. Lancet.

[23] Karlsson B, Jokura H, Yang HC (2019). The NASSAU (new assessment of cerebral arteriovenous malformations yet unruptured) analysis: are the results from the ARUBA trial also applicable to unruptured arteriovenous malformations deemed suitable for gamma knife surgery?. Neurosurgery.

[24] Liščák R, Vladyka V, Simonová G, Janoušková L, Vymazal J, Kondziolka D (2002). The obliteration rate of AVM after Gamma Knife Radiosurgery. Radiosurgery.

[25] Yahya S, Heyes G, Nightingale P, Lamin S, Chavda S, Geh I (2017). Linear accelerator radiosurgery for arteriovenous malformations: updated literature review. J Clin Neurosci.

[26] Ding D, Yen CP, Xu Z, Starke RM, Sheehan JP (2014). Radiosurgery for low-grade intracranial arteriovenous malformations. J Neurosurg.

[27] Ding D, Starke RM, Kano H, Lee JYK, Mathieu D, Pierce J (2017). Stereotactic radiosurgery for Spetzler-Martin Grade III arteriovenous malformations: an international multicenter study. J Neurosurg.

[28] Patibandla MR, Ding D, Kano H, Xu Z, Lee JYK, Mathieu D (2018). Stereotactic radiosurgery for Spetzler-Martin Grade IV and V arteriovenous malformations: an international multicenter study. J Neurosurg.

[29] Friedman WA, Bova FJ, Mendenhall WM (1995). Linear accelerator radiosurgery for arteriovenous malformations: the relationship of size to outcome. J Neurosurg.

[30] Kano H, Kondziolka D, Flickinger JC, Yang HC, Flannery TJ, Awan NR (2012). Stereotactic radiosurgery for arteriovenous malformations, Part 3: outcome predictors and risks after repeat radiosurgery. J Neurosurg.

[31] Singfer U, Hemelsoet D, Vanlangenhove P, Martens F, Verbeke L, Van Roost D (2017). Unruptured brain arteriovenous malformations: primary ONYX embolization in ARUBA (A randomized trial of unruptured brain arteriovenous malformations)-eligible patients. Stroke.

[32] Rammos SK, Gardenghi B, Bortolotti C, Cloft HJ, Lanzino G (2016). Aneurysms associated with brain arteriovenous malformations. AJNR Am J Neuroradiol.

[33] Kemeny AA, Dias PS, Forster DM (1989). Results of stereotactic radiosurgery of arteriovenous malformations: an analysis of 52 cases. J Neurol Neurosurg Psychiatry.

